# Activity of Hydrophilic, Biocompatible, Fluorescent, Organic Nanoparticles Functionalized with Purpurin-18 in Photodynamic Therapy for Colorectal Cancer

**DOI:** 10.3390/nano14191557

**Published:** 2024-09-26

**Authors:** Rayan Chkair, Justine Couvez, Frédérique Brégier, Mona Diab-Assaf, Vincent Sol, Mireille Blanchard-Desce, Bertrand Liagre, Guillaume Chemin

**Affiliations:** 1 University Limoges, LABCiS, UR 22722, 87000 Limoges, France; rayan.chkair@unilim.fr (R.C.); frederique.bregier@unilim.fr (F.B.); vincent.sol@unilim.fr (V.S.); bertrand.liagre@unilim.fr (B.L.); 2 University Bordeaux, CNRS, Bordeaux INP, ISM (UMR5255), Bat A12, 351 Cours de la Libération, 33405 Talence, France; justine.couvez@u-bordeaux.fr; 3 Doctoral School of Sciences and Technology, Lebanese University, Hadath, Beirut 21219, Lebanon; mdiabassaf@ul.edu.lb

**Keywords:** colorectal cancer, photodynamic therapy, purpurin-18, nanocarriers, FONPs, apoptosis

## Abstract

Photodynamic therapy (PDT) is a clinically approved, non-invasive therapy currently used for several solid tumors, triggering cell death through the generation of reactive oxygen species (ROS). However, the hydrophobic nature of most of the photosensitizers used, such as chlorins, limits the overall effectiveness of PDT. To address this limitation, the use of nanocarriers seems to be a powerful approach. From this perspective, we have recently developed water-soluble and biocompatible, fluorescent, organic nanoparticles (FONPs) functionalized with purpurin-18 and its derivative, chlorin p6 (Cp6), as new PDT agents. In this study, we aimed to investigate the induced cell death mechanism mediated by these functionalized nanoparticles after PDT photoactivation. Our results show strong phototoxic effects of the FONPs[Cp6], mediated by intracellular ROS generation, and subcellular localization in HCT116 and HT-29 human colorectal cancer (CRC) cells. Additionally, we proved that, post-PDT, the FONPs[Cp6] induce apoptosis via the intrinsic mitochondrial pathway, as shown by the significant upregulation of the Bax/Bcl-2 ratio, the activation of caspases 9, 3, and 7, leading poly-ADP-ribose polymerase (PARP-1) cleavage, and DNA fragmentation. Our work demonstrates the photodynamic activity of these nanoparticles, making them promising candidates for the PDT treatment of CRC.

## 1. Introduction

Colorectal cancer (CRC) is one of the most prevalent malignancies worldwide, ranking second in terms of cancer-related deaths and third in terms of incidence in 2022, according to the World Health Organization (WHO). By 2035, it is estimated that CRC incidence will increase by 80%, resulting in approximately 2.4 million cases and 1.3 million deaths globally [1,2]. Once diagnosed, cancer patients receive several treatment modalities, alone or in combination, depending on the stage and location of the tumor. Conventional therapies that are commonly used include surgery, radiotherapy, chemotherapy, and immunotherapy. These therapies are very effective in the early stages, but their efficacy becomes very limited in the later stages. Besides this limitation, conventional treatment is often associated with severe side effects, cancer recurrence, and resistance [3]. Hence, it has become vital to develop new therapeutic strategies that selectively target tumor sites while improving patients’ quality of life.

Photodynamic therapy (PDT) has emerged as a potential localized treatment, showing promising outcomes with solid tumors (such as gastrointestinal, prostate, and bladder cancers) [4,5], with improved tumor selectivity and minimal adverse effects compared to conventional cancer therapies [6]. Moreover, phase I and II clinical trials showed the clinical efficacy of PDT in CRC as a successful approach [7]. The mechanism of action relies on the dynamic interaction between three non-toxic components: a photosensitizer (PS), light with a suitable wavelength that activates the PS, and molecular oxygen. Once administered, the PS preferentially accumulates within the tumor and converts from its ground state (PS_0_) to an excited singlet state (PS_1_), under tumor illumination. The latter is unstable: it can return to the ground state by fluorescence emission or generate heat via internal conversion. However, this excited state can generate a more stable excited triplet state (T_1_) through an intersystem crossing. By this triplet state, the PS can undergo two kinds of photochemical reactions, leading to the production of reactive oxygen species (ROS), which are highly reactive and can damage biomolecules, triggering PDT-induced cancer cell death. In the case of type I reactions, the triplet state reacts with the cellular compounds by electron transfer, producing free radicals that, in the presence of molecular oxygen, generate hydroxyl radical (•OH), superoxide anion radical (O_2_^•−^), and hydrogen peroxide (H_2_O_2_). On the other hand, the type II reactions occur when the triplet state interacts directly with the molecular oxygen, resulting in singlet oxygen (^1^O_2_) generation [8].

Like any therapeutic option, PDT presents some limitations, such as the hydrophobicity of most of the traditional PSs used in the literature, PS aggregation occurring in biological environments, and selective cellular uptake [6]. These factors reduce the PDT’s clinical efficiency, limiting its overall therapeutic potential. To address these limitations, and thanks to the development of nanotechnology, especially in the context of CRC treatment, PS delivery efficiency may be improved by the encapsulation of the PS within nanoparticles (NPs), providing enhanced water solubility, tumor selectivity through passive or active approaches, and the reduction of toxic effects [7,9]. For this purpose, a wide range of nanocarriers were used in the literature, mainly categorized into organic and inorganic NPs [10,11]. The main advantage of organic NPs is their high biocompatibility, as they can be easily modified to control their physical and chemical characteristics [12]. Overall, the preclinical studies demonstrate the benefits of using nano-encapsulated PSs in the CRC model [7,13,14].

Among chlorins, purpurin-18 (Pp-18), is one of the most commonly used PSs in the literature. With an absorption maximum of 700 nm and a high singlet oxygen quantum yield, Pp-18 has been shown as a strong inducer of PDT-mediated cell death [15,16,17]. However, its clinical application is limited due to its high hydrophobicity. Thanks to its chemical structure that offers the possibility of different structural modifications, several approaches have been reported to improve its physicochemical properties, such as conjugation with peptides [18], gold nanoparticles [19], polyethylene glycol (PEG) [20], and sugars [21]. In addition, targeted nanoparticles as drug delivery systems has become one of the most promising strategies for CRC cancer therapy [22].

In this context, we have recently developed a new nano-formulation, based on dedicated soft fluorescent organic nanoparticles (FONPs) as nanocarriers of Pp-18 and its derivative chlorin p6 (Cp6). The functionalized FONPs, hereafter referred to as FONPs[Cp6], represent potential biocompatible nanoparticles for PDT treatment in CRC [23]. We stress that targeted nanoparticles The FONPs were prepared from an equimolar mixture of citric acid and diethylenetriamine (DETA) via polycondensation reactions using precise synthesis protocols (Scheme S1). The dehydration can be achieved either by heating a water solution under microwave activation [24] or by the dry heating of the diethlylenetriamonium citrate salt at 200 °C [23]. Importantly, controlled reaction conditions need to be implemented to avoid the carbonization and formation of the graphitic region. As a result, the FONPs had dry diameters of approximately 10–15 nm (Figure S1) and showed high water-solubility (>200 g/L) and biocompatibility [23]. Aqueous FONP solutions strongly absorb in the near UV-region (λ_abs_ ^max^ = 360 nm) and generate bright blue fluorescence under excitation due to an endogenous chromophore formed during the condensation process [23,24]. The FONPs showed a large number of CO_2_H and NH_2_ surface groups, which were responsible for their high-water solubility. These groups are of major interest for further functionalization using standard conjugation reactions. In order to implement such conjugations, the FONPs were activated either (i) with succinic acid [24], leading to organic nanoparticles enriched in CO_2_H surface groups, or (ii) with ethylene diamine [23] leading to organic nanoparticles enriched NH_2_ surface groups. In particular, the reaction of the latter with Pp18 resulted in the FONPs[Cp6] having a total PS loading (i.e., Cp6+ Pp18) of approximately 35 μmol.g^−1^. These nanoparticles retained good water solubility and showed the characteristic absorption bands of Cp6 and Pp18 in the visible region (Figure S2), with peaks at 420 nm (Soret Band), 500 nm, 560 nm, 660 nm, and 710 nm (Q bands). Interestingly, the FONPs[Cp6] showed deep-red emissions upon excitation in the visible region, as well as weak NIR emissions when excited at 720 nm (Figure S3). Moreover, the FONPs[Cp6] exhibited a high singlet oxygen generation quantum yield in aqueous media (0.72 upon excitation at 405 nm) [23].

The FONPs[Cp6] were found to show a strong in vitro phototoxicity against two CRC cell lines, HCT116 and HT-29, after illumination at 650 nm. Indeed, our previous results demonstrated the PDT potential of these new NPs with half-maximal inhibitory concentrations (IC_50_) of 0.04 and 0.13 nmol/mL for the HCT116 and HT-29 CRC cell lines, respectively [23].

Based on these promising results, the present study aimed to investigate the cell death mechanism mediated by these FONPs. First, we showed an excellent safety profile of the FONPs against human CRC cell lines, HCT116 and HT-29. Then, we demonstrated that the intracellular accumulation and the subcellular localization of the FONPs[Cp6] induced a strong anticancer efficacy through ROS production. Consistent with other in vitro PDT studies, we validated that once photoactivated, the FONPs[Cp6] induced apoptosis via the intrinsic pathway.

## 2. Results

### 2.1. In Vitro Safety of FONPs

**Figure 1:**
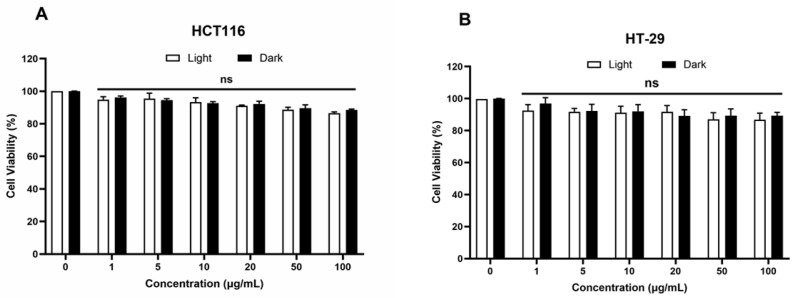
Phototoxic effects of FONPs on human CRC cell lines. HCT116 (**A**), and HT-29 (**B**) cells were seeded in 96-well plates for 24 h, then cells were treated or not with FONPs at different concentrations for 24 h. Cells were illuminated or not with red light (650 nm, 38 mW/cm^2^). The cytotoxic effects were then monitored 48 h following illumination by MTT assay. Data are shown as mean ± SEM (n = 3); ns: not significant relative to the control group.

Our previous study demonstrated the in vitro phototoxicity of FONPs functionalized with Pp18 and its derivative Cp6 (FONPs[Cp6]) [23], so we aimed first to investigate the FONPs safety as drug delivery nanocarriers in human CRC cell lines, HCT116 and HT-29. For this aim, the cells were treated with the FONPs at 1 to 100 μg/mL concentrations. Then, the cells were exposed to red light (650 nm), and the phototoxic effects were monitored for 48 h post-illumination using an MTT assay. Our results showed no significant reduction in cell viability when the FONPs were exposed to light or kept in the dark, even at a concentration of 100 μg/mL in both cell lines (Figure 1). These results further complement the previous findings of the absence of the dark cytotoxicity of the FONPs[Cp6], suggesting the good safety of the FONPs in human CRC cell lines.

### 2.2. Cellular Internalization and Localization of FONPs[Cp6]

**Figure 2:**
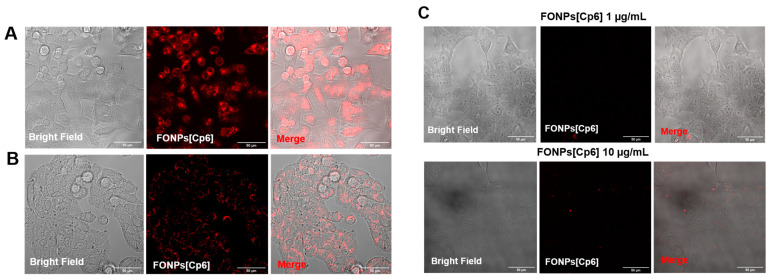
Cellular internalization of FONPs[Cp6] on HCT116 (**A**), HT-29 (**B**), and HEK-293 (**C**) cell lines. Cells were grown for 24 h in chamber slides coated with a type I collagen (3 mg/mL) with acetic acid (20 mM) gel. Cells were then treated with FONPs[Cp6] at IC_50_ concentrations for HCT116 and HT-29 and 1 or 10 µg/mL for HEK-293 cells. After 24 h, red fluorescence was assessed by confocal microscopy (Zeiss LSM880 confocal microscopeJena, Germany). Co-localization was analyzed using the ImageJ software. White scale bars represent 50 µm.

For confocal microscopy analyses, the HCT116 and HT-29 CRC cell lines were treated with the FONPs[Cp6] at IC_50_ concentrations already calculated: 1.40 and 3.86 µg/mL for the HCT116 and HT-29 cells, respectively, (corresponding to 0.04 nmol/mL and 0.13 nmol/mL of active PSs, respectively). The FONPs[Cp6] exhibited a deep-red or infrared NIR fluorescence [23], so their cellular uptake could be monitored by confocal microscopy when excited typically at 520 nm. The red fluorescence observed in the cytoplasm of the HCT116 and HT-29 CRC cells indicated the cellular internalization of the NPs in both of the cell lines (Figure 2 and Figure S5). Interestingly, compared to the noncancerous cell line HEK-293, we observed higher cellular accumulation within the HCT116 and HT-29 CRC cells, with a negligible uptake in HEK-293 cells, even at 10 µg/mL (Figure 2C). These results show that our NPs were preferentially internalized into CRC cells.

**Figure 3:**
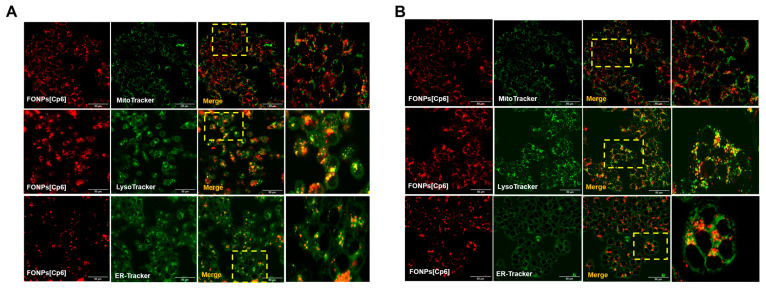
Cellular localization of FONPs[Cp6] on HCT116 (**A**) and HT-29 (**B**) CRC cell lines. Cells were seeded in chamber slides for 24 h prior to exposure to FONPs[Cp6] at IC_50_ concentrations. After 24 h, cells were co-treated with MitoTracker, LysoTracker, or ER-Tracker organelle probes. The images on the right of each panel correspond to the zoomed-in merged images (yellow box). Localization was determined by confocal microscopy, and co-localization was assessed using the ImageJ software. Scale bar = 50 µm.

PDT-induced cell death depends on the intracellular localization of the PS. Thus, after confirming the cellular uptake, we looked to determine the subcellular localization of the FONPs[Cp6] in the CRC cells. For this aim, the cells were co-treated with MitoTracker, LysoTracker, or Endoplasmic Reticulum-Tracker (ER-Tracker) organelle probes. The confocal fluorescence images in Figure 3 showed that the FONPs[Cp6] were distributed in all organelles, including the mitochondria, lysosomes, and ER (as indicated by yellow fluorescence in merged images), demonstrating the co-localization of the FONPs[Cp6] in both of the cell lines.

### 2.3. ROS Generation

**Figure 4:**
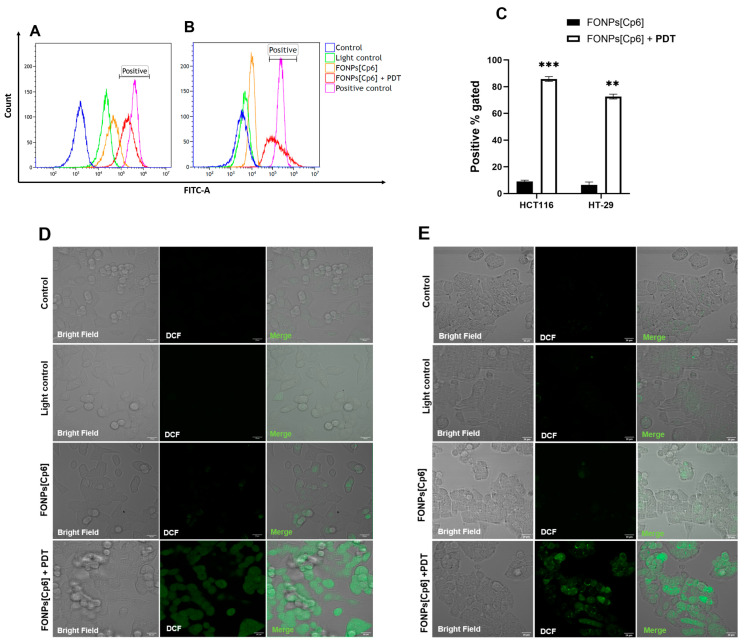
ROS generation by the FONPs[Cp6] in HCT116 (**A**,**C**,**D**) and HT-29 (**B**,**C**,**E**) CRC cell lines. For all experiments, cells were grown for 24 h. Then, cells were treated with the FONPs[Cp6] at IC_50_ concentrations and illuminated or not. Intracellular ROS generation was quantified by flow cytometry using DCFDA staining directly after PDT. An increased fluorescence intensity, resulting from increased 2′,7′-dichlorofluorescein (DCF) formation, causes a shift to the right. Hydrogen peroxide (H_2_O_2_) was used as a positive control (**A**,**B**). Results of flow cytometry analyses are shown in (**C**). The confocal microscopy images of DCFDA-labeled HCT116 (**D**) and HT-29 (**E**) CRC cells were captured immediately after PDT illumination. Scale bar = 20 µm. Data are represented as mean ± SEM (n = 3). ** *p* < 0.01 and *** *p* < 0.001, relative to the control.

The PDT-induced cell death mechanism involves intracellular ROS generation. Therefore, ROS levels were quantified using DCFDA staining after PDT illumination. Flow cytometry analyses revealed that the photoactivation of the FONPs[Cp6] enhanced cellular ROS generation in both cell lines (Figure 4A,B). Moreover, our results showed a greater ROS production, with 85.7% of positive gated in the HCT116 CRC cells compared to 72.6% in the HT-29 CRC cells (Figure 4C). In both the cell lines, the FONPs[Cp6] induced a limited ROS generation in the dark, with 9% and 6.4% of positive gated in the HCT116 and the HT-29 CRC cells, respectively.

These results were further confirmed by confocal microscopy, using the DCFDA as an ROS indicator. The confocal fluorescence images indicated a strong green fluorescence within the cells after the photoactivation of the FONPs[Cp6] compared to the control in both the cell lines (Figure 4D,E). Similarly, the HCT116 CRC cells showed an increased green fluorescence compared to the HT-29 CRC cells, demonstrating an enhanced ROS generation in the HCT116 CRC cells. Together, these data demonstrate that the photoactivation of the FONPs[Cp6] induces intracellular ROS generation in both the CRC cell lines.

### 2.4. Apoptotic Cell Death Induction

**Figure 5:**
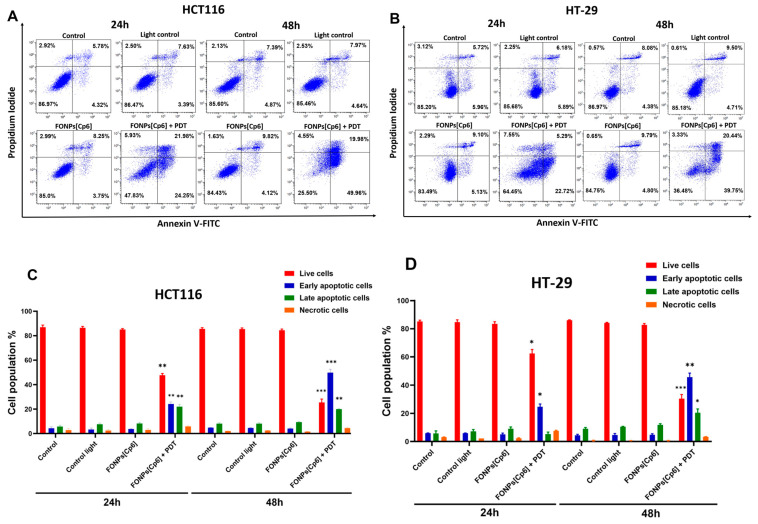
Apoptosis effects of the FONPs[Cp6] on HCT116 (**A**,**C**) and HT-29 (**B**,**D**) CRC cells. Cells were grown 24 h before exposure or not to the FONPs[Cp6] at IC_50_ concentrations. Then, cells were illuminated or not. Twenty-four and forty-eight hours after PDT illumination, cells were stained with Annexin V-FITC and PI, and cell apoptosis was measured by flow cytometry. (**A**,**B**) The results are represented in a scatter plot as four quadrants: living (lower left), early apoptotic (lower right), late apoptotic (upper right), and necrotic (upper left) cells. (**C**,**D**) The percentage of each cell population is expressed as mean ± SEM (n = 3). A t-test was used to compare each population in the treated group to its corresponding population in the control. * *p* < 0.05; ** *p* < 0.01, and *** *p* < 0.001, relative to the control.

In order to investigate the cell death mechanism induced by the FONPs[Cp6], Annexin V-FITC and PI dual staining were performed to assess the level of apoptosis 24 and 48 h post-illumination by flow cytometry. The early apoptotic cells could only retain Annexin V-FITC and were placed in the lower right quadrant. However, due to increased cell permeability, the late apoptotic cells could retain Annexin V-FITC and PI and were placed in the upper right quadrant. As shown in Figure 5A,C, in the HCT116 CRC cells, the control, light control, and the FONPs[Cp6] exhibited high cellular viabilities of 86.97%, 86.47%, and 85.00%, respectively, at 24 h. Similarly, 85.60%, 85.46%, and 84.43% of the population of the HCT116 CRC cells were viable after exposure to the control, light control, and the FONPs[Cp6], respectively, at 48 h. However, 24 h after the FONPs[Cp6] photoactivation, the rate of early and late apoptosis both increased significantly to 46.23%, while the percentage of viable cells decreased to 47.83%. Importantly, 48 h post-PDT, the rate of apoptosis continued to rise to 69.94%, with only 25.50% being viable cells. Similar results were observed with the HT-29 CRC cells: 85.20%, 85.68%, and 83.49% of the cell population were viable after exposure to the control, light control, and the FONPs[Cp6], respectively, at 24 h. After 48 h, 86.97%, 85.18%, and 84.75% of the control, light control, and the FONPs[Cp6], respectively, remained mostly viable. Twenty-four hours after illumination, the FONPs[Cp6] induced a significant increase in the rate of apoptosis to 28.01%, with 64.45% being viable cells. Moreover, after 48 h of photoactivation, the rate of apoptosis increased further to reach 60.19%, while the percentage of viable cells decreased to 36.48% (Figure 5B,D). Together, these findings provide evidence that the photoactivation of the FONPs[Cp6] induces a significant increase in the rate of early and late apoptosis, thus confirming that apoptosis was one of the major induced cell death mechanisms.

### 2.5. PDT-Induced Apoptosis Mechanism

**Figure 6:**
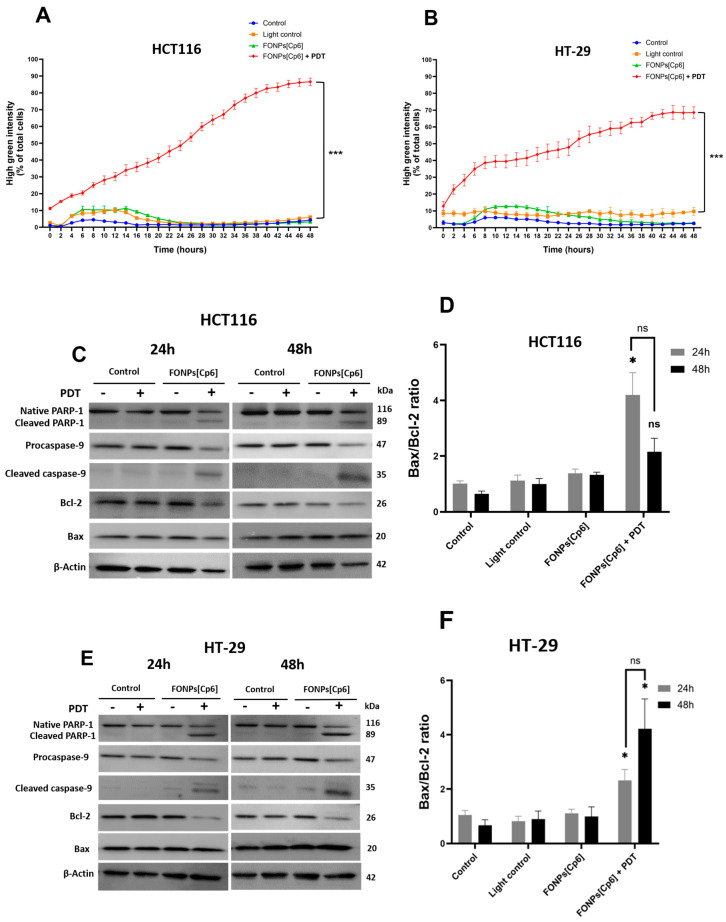
Effects of photoactivation of the FONPs[Cp6] on caspases-3/7 activity and protein expression in HCT116 (**A**,**C**,**D**) and HT-29 (**B**,**E**,**F**) CRC cells. Cells were cultured for 24 h, then treated with the FONPs[Cp6] at IC_50_ concentrations for 24 h, then illuminated. (**A**,**B**) Quantification of caspases-3/7 activity. Directly after PDT of cells co-treated with caspases-3/7 green reagent, fluorescence over time was monitored by Incucyte S3 live-cell imaging system. The ratio of green fluorescent cells to total cells was quantified using IncuCytesoftware version 2023A Rev1). (**C**–**F**) The expression of the key apoptotic proteins was assessed by western blotting 24 and 48 h after illumination. β-actin served as a loading control. Bands were quantified by densitometry using ImageJ software, and the Bax/Bcl-2 ratio was then calculated relative to β-actin. * *p* < 0.05 and *** *p* < 0.001, relative to the control; ns: not significant.

Caspases are known for their crucial role in the initiation and execution of apoptosis. Hence, to confirm the apoptotic cell death mechanism observed after the photoactivation of the FONPs[Cp6], the level of the caspases-3/7 activity, the main apoptosis executioner, was measured using the Incucyte S3 live-cell imaging system. For this purpose, the cells were labeled with caspases-3/7 green reagent immediately after PDT, and their fluorescence was monitored for 48 h. As observed in Figure 6A, the photoactivation of the FONPs[Cp6] in the HCT116 CRC cells induced a significant increase in the percentage of activated caspases-3/7 in a time-dependent manner, with 86.6% compared to 6.1% in the light control, at 48 h post-illumination. Similar results were obtained with the HT-29 CR+C cells, with a significant increase in caspase-3/7 activity level to 68.5%, compared to 9.6% in the light control (Figure 6B).

To further confirm the apoptotic cell death, we conducted western blotting to assess the protein expression of key apoptotic-related markers. We focused mainly on poly-ADP-ribose polymerase (PARP-1), which is a substrate of caspase-3, caspase-9 (initiator caspase), Bax (pro-apoptotic), and Bcl-2 (anti-apoptotic) protein expression, 24 and 48 h following photoactivation. In the HCT116 CRC cells, our results show that the photoactivation of the FONPs[Cp6] induced a significant increase in the Bax/Bcl-2 ratio, by 4.19-fold compared to the control. After 48 h, this ratio decreased to 2.1-fold. These observations were correlated with caspase-9 activation, leading to its cleavage, when the FONPs[Cp6] were photoactivated. Subsequently, the activation of caspase-3 (Figure 6C, D) resulted in PARP-1 cleavage (inactivation), associated with a decrease in the expression of the native PARP-1, 24 and 48 h after the FONPs[Cp6] illumination. Similar results were observed in the HT-29 CRC cells: the Bax/Bcl-2 ratio was significantly increased by 2.31- and 4.1-fold at 24 and 48 h, respectively, compared to the control. The increase in the Bax/Bcl-2 ratio was correlated with the activation of caspase-9, and its cleavage 24 and 48 h following PDT. Furthermore, the PARP-1 cleavage was mainly observed 24 and 48 h after the FONPs’[Cp6] photoactivation (Figure 6E,F).

**Figure 7:**
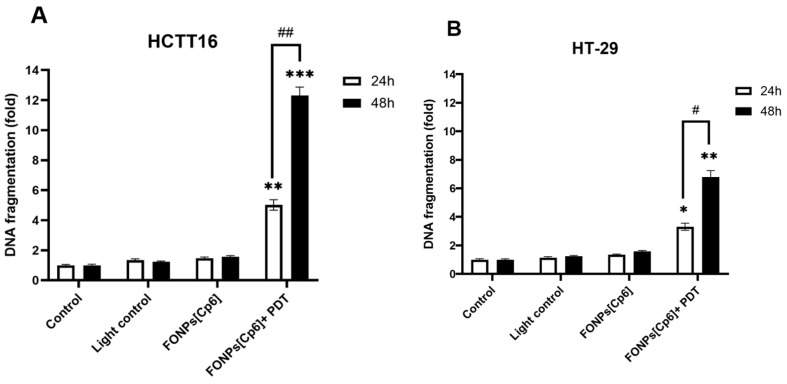
Effects of photoactivation of the FONPs[Cp6] on DNA fragmentation in HCT116 (**A**) and HT-29 (**B**) CRC cells. Cells were grown for 24 h before being treated or not with the FONPs[Cp6] at IC_50_ concentrations. After 24 h of incubation, cells were then illuminated or not. DNA fragmentation 24 and 48 h post-illumination was quantified from cytosol extracts by ELISA assay. The results are expressed as n-fold compared to the control. Data are expressed as mean ± SEM (n = 3). * *p* < 0.05, ** *p* < 0.01, and *** *p* < 0.001, compared to the control, or # *p* < 0.05 and ## *p* < 0.01, compared to 24 h data.

In the absence of PARP-1 repair, DNA is cleaved by endonucleases. To evaluate the apoptotic process at the later stages and examine the nuclear changes induced by the FONPs[Cp6], DNA fragmentation was performed 24 and 48 h after PDT using an ELISA assay. In the HCT116 CRC cells, the results indicate that the FONPs[Cp6], after PDT, induced a strong increase in DNA fragmentation by 5.2- and 12.3-fold, at 24 and 48 h, respectively, compared to the control (Figure 7A). As expected, the FONPs[Cp6] in the dark induced very limited DNA fragmentation of 1.3- and 1.5-fold at 24 and 48 h, respectively, compared to the control. The same results were observed in the HT-29 CRC cells: the FONPs[Cp6] photoactivation induced a significant increase in DNA fragmentation by 3.3- and 6.3-fold at 24 and 48 h, respectively, when compared to the control. The FONPs[Cp6] in the dark showed no significant DNA fragmentation, with 1.3- and 1.5-fold changes at 24 and 48 h, respectively (Figure 7B).

Taken together, these results demonstrate that the FONPs[Cp6] photoactivation induced apoptosis in the human CRC cell lines via the intrinsic pathway.

## 3. Discussion

Presently, extensive efforts have been made to overcome the conventional PDT limitations, particularly through nanocarrier platforms. The use of NPs seems to be an attractive strategy for improving traditional PSs’ hydrophilicity and accumulation within tumor sites. Interestingly, targeted NPs are a novel class of NPs that were developed by conjugating with ligands or other molecules to facilitate cancer cell recognition. These NPs are widely used in the literature to treat CRC [22]. In a previous study [23], we investigated the anticancer efficiency of a new nano-formulation (FONPs[Cp6]) of hydrophobic PS, Pp-18, and its derivative, Cp6, on human CRC cell lines (HCT116 and HT-29). Our previous work demonstrated that the FONPs[Cp6] combine several attractive characteristics, including high water solubility and stability, a high singlet oxygen quantum yield, and deep-red or NIR fluorescence [23]. So, after confirming the photodynamic activity of these functionalized NPs, the main objective of the present study was to understand the induced cell death mechanism that contributes to anticancer effects. First, we evaluated the in vitro safety of the FONPs, and we showed that these nanocarriers were extremely safe on the human CRC cell lines and were found to selectively accumulate within the cancer cells, compared to noncancerous HEK-293 cells. These results confirmed a previous study using the FONPs on glioblastoma cells, in which they were shown to preferentially accumulate in glioblastoma cells, mainly by caveolin and lipid rafts endocytosis [24].

On the other hand, the subcellular localization of PSs plays a critical role in determination of the extent of the cellular damage and the mechanisms involved in cell death. Our nanocarriers were found to accumulate in the cytoplasm of both the cell lines, but not in the nucleus. These results correlate with a previous study, in which Pp-18 was loaded into solid lipid nanoparticles (SLNs) and evaluated against HeLa and A549 lines [25]. Moreover, our confocal fluorescence images showed that the FONPs[Cp6] seemed to localize in lysosomes, mitochondria, and ER organelles. Similarly, a research group found that the conjugation of Pp-18 with a polyethylene glycol (PEG) spacer led to improved accumulation in the mitochondria, lysosomes, and ER in various human cancer cells [20].

Apoptosis is the most commonly described cell death after PDT, mainly due to ROS generation [26]. The intracellular ROS generation results showed that the FONPs[Cp6] exhibited high photoactivity, with 85.7% and 72.6% of ROS production in the HCT116 and the HT-29 CRC cell lines, respectively. When compared to Liu et al.’s work [27], in which they obtained water dispersible organic nanoparticles (WSONs) from the nanoprecipitation in water of hydrophobic chlorin derivatives, the FONPs[Cp6] in the present study seem to have a higher photodynamic efficiency. Liu et al.’s study highlighted that WSONs obtained from purpurin18 methyl ester (pu18ME) induce mitochondrial apoptotic cell death, with singlet oxygen generation of less than 65% in HeLa cell lines [27]. The use of a carbon monoxide (CO) controllable release system has emerged as an efficient anticancer therapy [28]. An interesting approach, reported by Wu et al., is the PDT-driven CO release system, based on Cp6, using nanogels. They showed that, when exposed to light irradiation, this system induces synergistic anticancer effects shown by the high CO and singlet oxygen (^1^O_2_) generation, demonstrating a strong photodynamic efficiency in both in vitro and in vivo breast cancer models [29].

Regarding the apoptotic cell death mechanism, we validated that the FONPs[Cp6] induce apoptosis via the intrinsic pathway. As localized in the mitochondria, the photoactivation of the FONPs[Cp6] induces ROS generation that induces damage to the mitochondrial membrane, followed by an increase in the Bax/Bcl-2 ratio, leading to the subsequent activation of caspase-9. Once activated, caspase-9 cleaves and activates the executioner caspases 3 and 7, leading to PARP-1 cleavage and DNA fragmentation. Consistent with our results, a study reported that the photoactivation of Cp6 induces apoptosis and autophagy via ROS generation in human CRC SW620 cells [17]. Furthermore, Bharathiraja et al. demonstrated that Cp6 conjugated with silica nanoparticles enhanced cellular uptake and induced apoptosis by intracellular ROS generation in breast cancer cells [30]. An in vivo study on hepatocellular carcinoma, using Pp-18 conjugated with gold NPs, showed that PDT-irradiation induced caspase-3 activation and DNA fragmentation [31].

Accumulating evidence suggests that autophagy is triggered after PDT due to ROS generation. In fact, autophagy seems to play a controversial role in the PDT context. Several studies have demonstrated that applying an autophagy inhibitor can enhance PDT-induced cell death [17,32]. However, other studies showed that autophagy inhibition significantly decreased PDT-induced cell death in breast cancer cells [33]. In our work, we proved that the FONPs[Cp6] were localized in the lysosomes and ER, so further investigations of the role of autophagy in the induced apoptotic cell death could be useful. Indeed, the use of 3D cell spheroid models of CRC that imitate the tumor microenvironment seems to be crucial to assessing the PDT potential of the FONPs[Cp6]. Additionally, the use of in vivo animal models (such as the subcutaneous xenograft mouse model of CRC) [34] to investigate these novel nanocarriers would be the ultimate goal of the present work.

## 4. Materials and Methods

### 4.1. Materials

The RPMI 1640 and RPMI 1640 red-phenol-free mediums were purchased from Gibco BRL (Cergy-Pontoise, France). The 3-(4,5-dimethylthiazol-2-yl)-2,5-diphenyltetrazoliumbromide (MTT), 2′,7′-dichlorofluorescein diacetate (DCFDA) kit, cell death detection enzyme-linked immunosorbent assay^PLUS^ (ELISA), and human anti-actin antibodies were acquired from Sigma-Aldrich (Burlington, MA, USA). The Poly-ADP-ribose polymerase (PARP), caspase-9, Bcl-2, and Bax antibodies, goat anti-rabbit IgG H&L horseradish peroxidase (HRP) secondary antibodies, and LysoTracker Green DND-26 were obtained from Cell Signaling Technology-Ozyme (Danvers, MA, USA). The MitoTracker Green FM, ER-Tracker™ Green, annexin V-FITC, propidium iodide (PI), and rabbit anti-mouse IgG-IgM H&L HRP secondary antibodies were obtained from Invitrogen-Thermo Fisher Scientific (Van Allen Way, Carlsbad, CA, USA).

### 4.2. Synthesis of the FONPs[Cp6]

The FONPs and the FONPs[Cp6] were prepared as described in ref [23]. Briefly, the ammonium citrate salt was first prepared by mixing equimolar amounts of diethylenetriamine (DETA) and citric triacid in pure water, then evaporating the water under vacuum to obtain a dry solid. The salt was subsequently heated at 200 °C for 30 min, leading to a brownish–yellowish crude material, which was washed with ethanol and then dried under vacuum. The bare FONPs were then enriched in NH_2_ surface groups by heating in ethylene diamine at 120 °C for 16 h [35]. Finally, the FONPs^NH^_2_ were reacted with Pp18 (Scheme S1, step 3) in a biphasic medium (H_2_O/toluene) under vigorous stirring and heating at 100 C to yield the FONPs[Cp6] after the separation of the aqueous phase, freeze-drying, and washing with CH_2_Cl_2_, then drying under a vacuum. The FONPs[Cp6] showed a size distribution ranging between 10 nm and 20 nm, giving a mean diameter of 12.9 nm, as indicated by transmission electron microscopy (Figure S1). A small number of larger nanoparticles (approximately 25 and 35 nm in diameter) were also detected [23]. Gel electrophoresis (Figure S4) indicated that both the FONPs^NH2^ and the FONPs[Cp6] were mainly positively charged.

### 4.3. Cell Lines Culture and Treatment

The human CRC HCT116 and HT-29 adherent cell lines were purchased from the American Type Culture Collection (ATCC—LGC Standards, Molsheim, France). We chose these human CRC cell lines because they are of different stages, enabling us to evaluate the possible resistance to our treatments: The HCT116 CRC line was isolated from a stage I colorectal carcinoma from an adult male. The HT-29 CRC line was derived from a stage II colorectal adenocarcinoma from a 44-year-old woman.

The cells were grown in an RPMI medium and cultured as previously mentioned [23], and the HEK cells were seeded at 2 × 10^4^ cells/cm^2^. The FONPs[Cp6] and FONP platform stock solutions were prepared in ultrapure water and diluted in a culture medium to prepare the working concentrations. The in vitro PDT protocol was performed as previously explained [23].

### 4.4. In Vitro Cytotoxicity Assay of FONPs

The cytotoxicity of the FONPs was assessed using the MTT assay, as described in [23], which reveals dehydrogenase activity in living cells. The cells were seeded in 96-well culture plates for 24 h before exposure or not to the compounds. After 24 h of incubation, the cells were illuminated with a 650 nm lamp (PhotoCure ASA, Oslo, Norway) as a light source at 38 mW/cm^2^. The cell viability percentage was then measured 48 h after illumination, by normalizing control cells.

### 4.5. Intracellular ROS Generation by the FONPs[Cp6]

ROS production was quantified using a cell-permeable fluorescent probe, 2′,7′-dichlorofluorescein diacetate (DCFDA), to determine the intracellular oxidative stress. To this end, the cells were seeded in 25 cm^2^ tissue culture flasks for 24 h before being treated or not with the FONPs[Cp6] at IC_50_ concentrations. After 24 h, the cells were stained with DCFDA (20 µM) at 37 °C. After 30 min of incubation, the cells were illuminated or not. ROS generation was assessed by flow cytometry (BD Biosciences, San Jose, CA, USA) directly after PDT. Hydrogen peroxide (H_2_O_2_) was used as a positive control (800 µM).

For confocal microscopy images, the cells were plated for 24 h in chamber slides (ibidi µ-Slide 8 well; Clinisciences, Gräfelfing, Germany) and coated with a type I collagen (3 mg/mL) and acetic acid (20 mM) gel. After this, the cells were treated with the FONPs[Cp6] at IC_50_ values. Then, after 24 h, DCFDA (20 µM) was added for 30 min, and the fluorescence images were taken under excitation of 488 nm at 40× magnification by confocal microscopy (Zeiss LSM880 confocal microscope).

### 4.6. Cellular Uptake and Localization of the FONPs[Cp6]

The cells were seeded for 24 h before treatment with the FONPs[Cp6] at IC_50_ concentrations in chamber slides coated as mentioned above to determine the cellular localization. Then, after 24 h, the cells were treated with MitoTracker Green FM (50 nM), ER-Tracker™ Green (500 nM), and LysoTracker Green DND-26 (50 nM) for 45 min, 30 min, and extemporaneously at 37 °C, respectively. The FONPs’[Cp6] localization was examined by confocal microscopy, using the FONPs[Cp6] fluorescence (excitation/emission: 520/665 nm) with MitoTracker fluorescence (490/516 nm), LysoTracker fluorescence (504/511 nm), and ER-Tracker fluorescence (504/511 nm). The co-localization was analyzed using the ImageJ software (version 1.52p).

### 4.7. Apoptotic Cell Death Induction by the FONPs[Cp6]

#### 4.7.1. Dual Staining Assay with Annexin V-FITC and PI

Annexin V-FITC/PI dual staining was performed to evaluate the apoptosis induction. The cells were treated with the FONPs[Cp6] for each cell line at IC_50_ concentrations and were illuminated or not. Twenty-four and forty-eight hours post-PDT, a total of 2.5 × 10^5^ cells/conditions were collected, then washed with PBS, centrifuged at 1500 rpm for 5 min, and incubated with 100 µL binding buffer (1X) with 5 µL annexin V-FITC and 1 µL of PI at 100 μg/mL for 15 min in the dark. After that, 200 µL of the binding buffer was added to the samples, and the percentage of apoptotic cells was analyzed by flow cytometry. Our data are represented in a scatter plot as four quadrants: living, early apoptosis, late apoptosis, and necrotic cells.

#### 4.7.2. DNA Fragmentation

A further analysis for cell death was performed using a Cell Death Detection ELISA^PLUS^ to detect DNA fragmentation. For this, the cells were treated or not with the FONPs[Cp6] at IC_50_ concentrations for 24 h, and then illuminated or not. Twenty-four and forty-eight hours after PDT, cytosolic extracts of 2 × 10^5^ cells/condition were obtained, and the amounts of mono- and oligonucleosomes produced from apoptotic cells were quantified, following the manufacturer’s protocol. The absorbance was measured at 405 nm, using a microplate reader (Thermoscientific MULTISKAN FC). The results are expressed as n-fold compared to the control.

#### 4.7.3. Quantification of Caspase-3/7 Activity

The amounts of activated caspases-3/7 were monitored using an IncucyteS3 system (Sartorius, Göttingen, Germany), a live-cell imaging device that allows fluorescence tracking over time. The cells were seeded in 96-well plates for 24 h before being treated or not with the FONPs[Cp6] at IC_50_ concentrations and illuminated. Then, the cells were labeled with caspases-3/7 green reagent (Sartorius) at 5 µM. The photos were taken every 2 h at ×20 magnification in phase contrast/green fluorescence. The Green Object Count/Phase Object Count ratio was quantified using Incucyte software (version 2023A Rev1) to determine the percentage of green fluorescent cells.

#### 4.7.4. Protein Expression by Western Blotting

Each cell line was treated with IC_50_ concentrations and illuminated. Twenty-four and forty-eight hours after PDT, the total cells of each condition were collected, and whole-cell lysate proteins were extracted using an RIPA lysis buffer (containing 50 mM HEPES, pH 7.5, 150 mM NaCl, 1% sodium deoxycholate, 1% NP-40, 0.1% SDS, and 20 mg/mL of aprotinin). The protein levels were quantified using the Bradford method. Briefly, the proteins (60–80 µg) were separated by electrophoresis on SDS-PAGE gels and then transferred to polyvinylidene difluoride (PVDF) membranes (Amersham Pharmacia Biotech, Saclay, France). After that, the membranes were incubated with primary antibodies against human apoptosis-related proteins, including PARP-1 (1:1000), caspase-9 (1:1000), Bcl-2 (1:1000), and Bax (1:1000). β-actin (1:5000) was used as a loading control. The membranes were then incubated with secondary antibodies, and bands were revealed using the Immobilon Western HRP Substrate (Merck, Darmstadt, Germany) and a G: BOX system (Syngene, Cambridge, UK). The bands were quantified by densitometry, using ImageJ software.

### 4.8. Statistical Analyses

All the data are presented as the mean ± standard error of the mean (SEM) of three independent experiments. The statistical analyses were performed using GraphPad Prism 9.0 by two-tailed unpaired Student’s *t*-tests. *p* values of * *p* < 0.05, ** *p* < 0.01, and *** *p* < 0.001 or # *p* < 0.05 and ## *p* < 0.01 were considered statistically significant.

## 5. Conclusions

In this study, we evaluated the anticancer efficiency of novel biocompatible FONPs that were functionalized with a hydrophobic PS (Pp-18 and its derivative Cp6) on human CRC cell lines (HCT116 and HT-29). We validated the in vitro safety of the FONPs as delivery nanocarriers for the hydrophobic PS. Interestingly, the FONPs[Cp6] showed a preferential internalization in the CRC cell lines. Furthermore, the confocal microscopy analysis confirmed that the FONPs[Cp6] were localized in the mitochondria, lysosomes, and ER, which are the desired targets for PDT. We can conclude that the FONPs[Cp6] exhibit strong photodynamic activity through ROS generation, resulting in induced apoptosis via the intrinsic pathway. Together, the in vitro results demonstrate the interesting anticancer efficiency of this nano-formulation against CRC. To further this study, the use of 3D cancer models could be the first step, followed by in vivo experiments to validate our findings.

## Data Availability

The data are contained within the article and Supplementary Materials.

## Supplementary Materials

The following supporting information can be downloaded at: https://www.mdpi.com/article/10.3390/nano14191557/s1. Synthesis and characterizations of FONPs[Cp6]. DAPI staining. Western blot analysis of the autophagy markers.

## Abbreviations

ATCC: American Type Culture Collection; Cp6: chlorin p6; CRC: colorectal cancer; DCFDA: 2′,7′-dichlorofluorescein diacetate; DNA: deoxyribonucleic acid; ELISA: enzyme-linked immunosorbent assay; ER-Tracker: endoplasmic reticulum tracker; FONPs: fluorescent organic nanoparticles; HEPES: 4-(2-hydroxyethyl)-1-piperazine ethane sulfonic acid; HEK-293 cells: human embryonic kidney-293 cells; H_2_O_2_: hydrogen peroxide; HRP: horseradish peroxidase; IC_50_: half-maximal inhibitory concentration; MTT: 3-(4,5-dimethylthiazol-2-yl)-2,5-diphenyltetrazolium bromide; NPs: nanoparticles; ^1^O_2_: singlet oxygen; •OH: hydroxyl radical; O_2_^•−^: superoxide anion; PARP-1: poly-ADP-ribose polymerase; PBS: phosphate-buffered saline; PDT: photodynamic therapy; PI: propidium Iodide; PS: photosensitizer; PVDF membrane: polyvinylidene fluoride membrane; Pp-18: purpurin-18; RIPA: radio immuno precipitation assay; ROS: reactive oxygen species; RPMI: Roswell Park Memorial Institute medium; SDSPAGE: electrophoresis in polyacrylamide gel containing sodium dodecyl sulfate; SEM: standard error of the mean.
