# Supplementary material for: Activity of Hydrophilic, Biocompatible, Fluorescent, Organic Nanoparticles Functionalized with Purpurin-18 in Photodynamic Therapy for Colorectal Cancer
Source: Nanomaterials (Basel). 2024 Sep 26;14(19):1557. doi: 10.3390/nano14191557 (PMC11478336; doi:10.3390/nano14191557)
Supplement: Supplementary file 1 [file nanomaterials-14-01557-s001.zip › nanomaterials-3184694-supplementary.pdf]

1. <u>Chemical synthesis of FONPs[Cp6]</u>

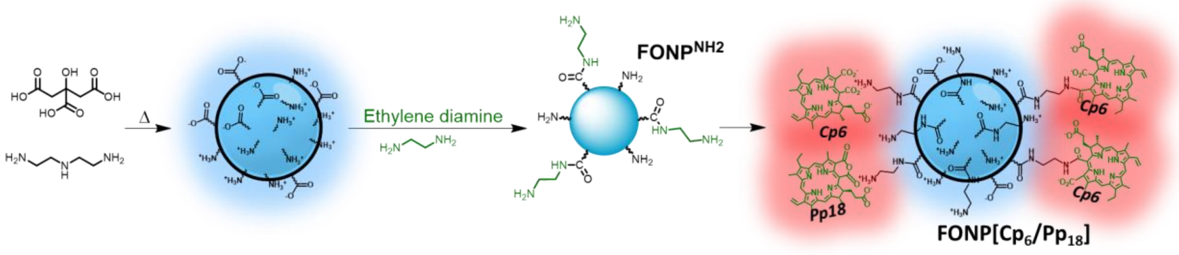

Scheme S1: Multistep synthesis of FONPs[Cp$_6$]. 1st step: synthesis of fluorescent organic nanoparticles FONPs by polycondensation of citric acid (1eq.) and diethylenetriamine (1eq.). 2nd step: enrichment in NH_2 surface groups to yield FONPs^{NH2}. 3rd step: Preparation of FONPs[Cp6] nanoformulation by reaction with Pp$_{18}$ [1]

2. <u>Transmission Electron Microscopy (TEM) characterization of FONPs[Cp6]</u>

Dry diameter of FONs[Cp6] was determined by TEM. A droplet of stock solution of FONPs^{Cp6} was deposited on positively charged carbon-membrane coated copper grids. After removing the droplet from the sample and air-drying the grid, a contrast agent (uranyl acetate 3wt%) was deposited onto the grids. Grids were air-dried before to be analysed on a Hitachi H7650 electron microscope (80 kV).

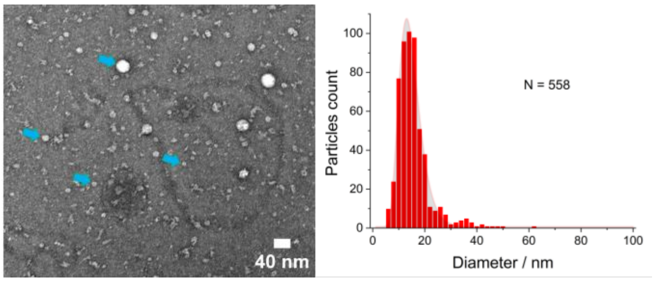

Figure S1: TEM images of FONPs[Cp6] (left). Size distribution of FONPs[Cp6] fitted with a log normal [1]

3. <u>Photophysical characterization of FONPs[Cp6]</u>

The absorption and the emission spectra were respectively recorded on a Jasco-V670 and a Fluoromax-3 spectrometers.

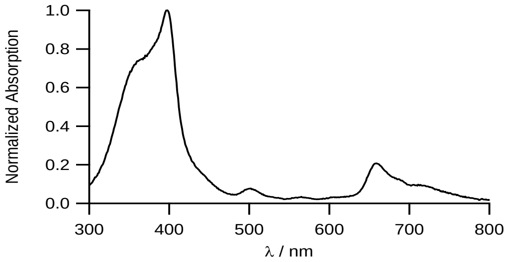

Figure S2: UV-Vis absorption spectrum of FONPs^{Cp6} in H_2O [1]

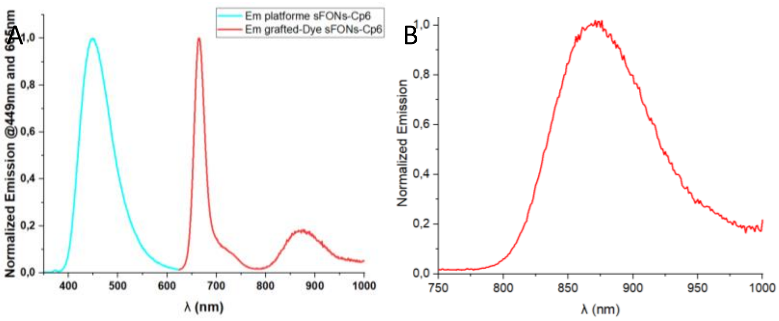

Figure S3: (A) Emission spectra of FONPs[Cp6] in PBS 1X under excitation at 330 nm (blue), 520 nm (red) (left). (B) Emission spectra of FONs[Cp6] in PBS under excitation at 710 nm (left). [1]

4. Gel Electrophoresis

Gel preparation: To prepare a 0.8wt% gel, 400mg of Agarose powder was dissolved in 50mL of aqueous TAE solution (pH-8) then microwave heated (1min at 600W). FONPs samples were deposited into wells at different concentrations (0.1g/L, 0.25g/L and 1g/L) then eluted (40V, 400mA, 90min).

1	2	3	4	5	6	7	8	9	10	11	12	13	14	15
X	$sFONs^{Cp6}$ 0.1g/L	X	$sFONs^{Cp6}$ 0.25g/L	X	$sFONs^{Cp6}$ 1g/L	X	$sFONs^{NH2}$ 1g/L	X	$sFONs^{NH2}$ 0.25g/L	X	$sFONs^{NH2}$ 0.1g/L	X	X	X

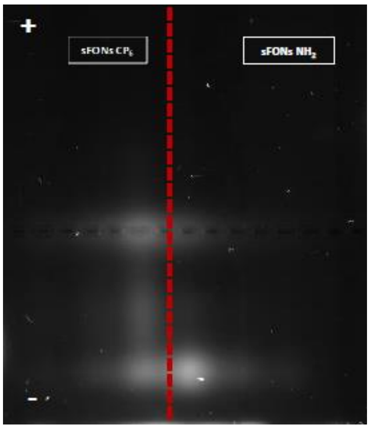

Figure S4. Gel Electrophoresis of FONPs[Cp6] (left) and FONPs ^{NH2} (right) at various concentrations. Top anode (+). Bottom Cathode (-). Visualization by fluorescence under excitation @ 365 nm

5. DAPI staining

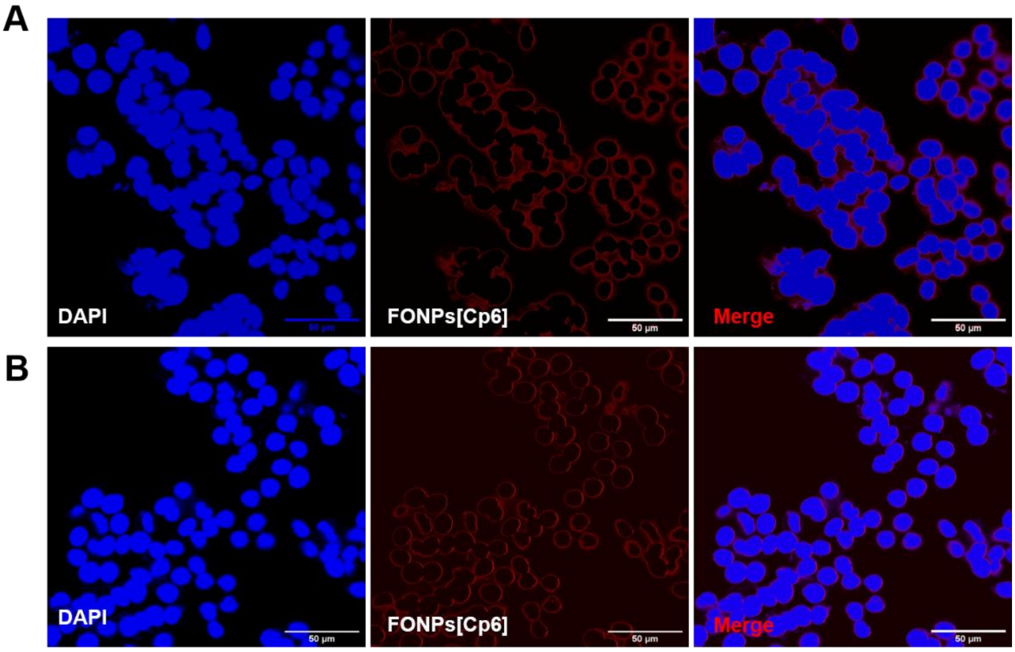

Figure S5. Cellular internalization of FONPs[Cp6] on HCT116 (A) and HT-29 (B). Cells were seeded in chamber slides for 24 h before exposure to FONPs[Cp6] at IC_{50} concentrations. After 24 h, nuclei were stained with DAPI (blue). The fluorescence was observed by confocal microscopy (Zeiss LSM880 confocal microscope). Co-localization was analyzed using the ImageJ software. Scale bars represent 50 µm.

6. Western-blot analysis of the different autophagy markers

A

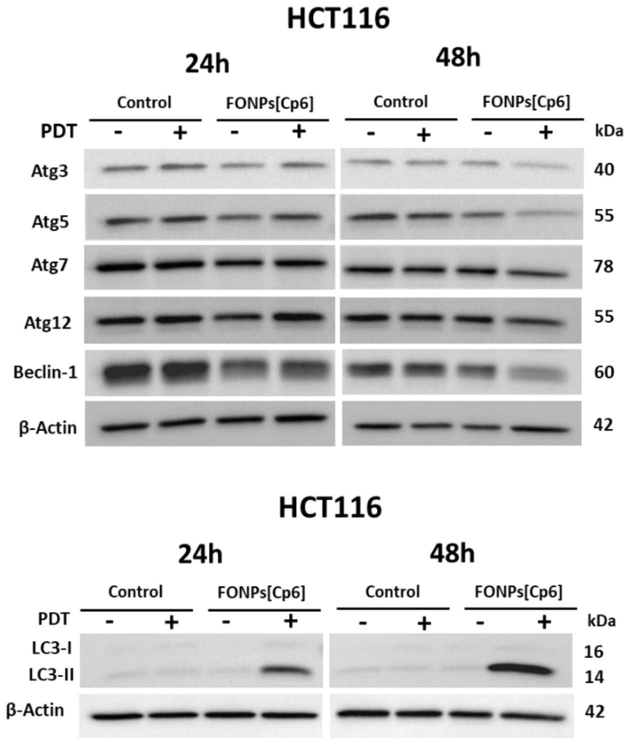

B

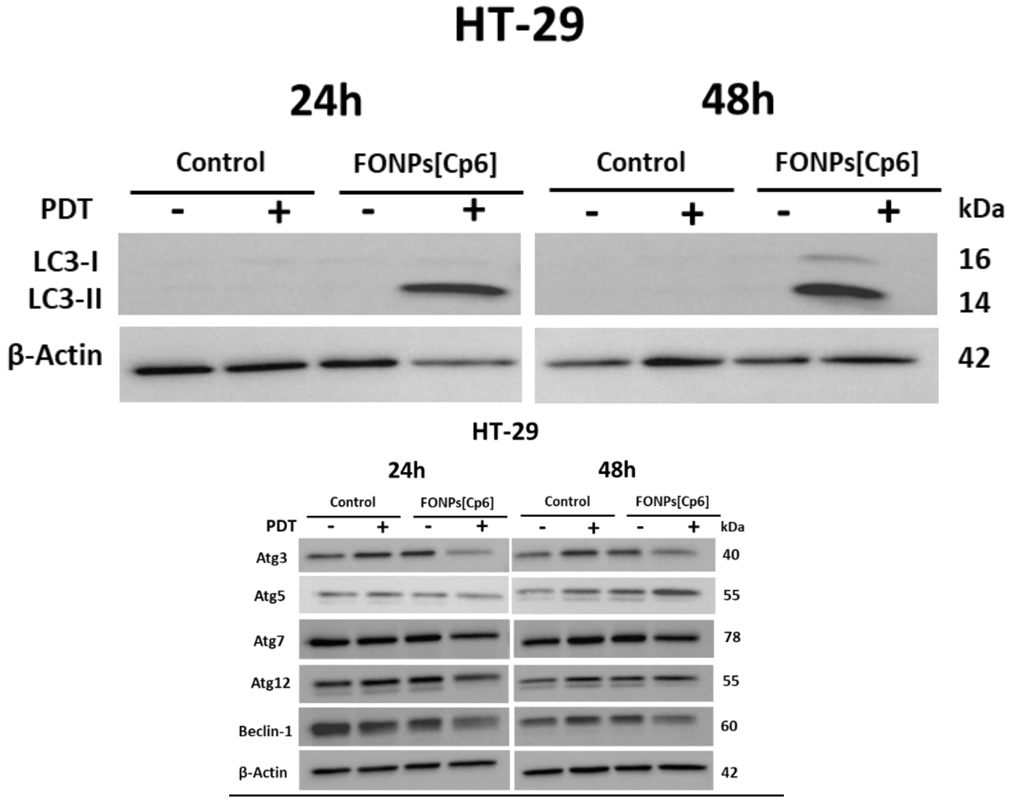

Figure S6. Effects of photoactivation of FONPs[Cp6] on protein expression in HCT116 (A)and HT-29 (B) CRC cells. Cells were cultured for 24 h, then treated with FONPs[Cp6] at IC_{50} concentrations for 24 h, then illuminated. The expression of the key autophagy-related proteins was assessed by Western blotting 24 and 48 h after illumination. β-actin serves as a loading control. Autophagy antibodies were acquired from Cell Signaling Technology—Ozyme (Saint-Quentin-en-Yvelines).

References

(1) Sasaki, I.; Brégier, F.; Chemin, G.; Daniel, J.; Couvez, J.; Chkair, R.; Vaultier, M.; Sol, V.; Blanchard-Desce, M. Hydrophilic Biocompatible Fluorescent Organic Nanoparticles as Nanocarriers for Biosourced Photosensitizers for Photodynamic Therapy. *Nanomaterials* **2024**, *14* (2), 216. https://doi.org/10.3390/nano14020216.
